# Effect of Multiparity on Pregnancy-Induced Islet Adaptation and Cellular Transdifferentiation

**DOI:** 10.1177/11795514251386124

**Published:** 2025-10-17

**Authors:** Vaibhav Dubey, Neil Tanday, Asif Ali, Andrei I. Tarasov, Peter R. Flatt, Nigel Irwin, R. Charlotte Moffett

**Affiliations:** 1 Centre for Diabetes, Schools of Biomedical Sciences and Pharmacy & Pharmaceutical Sciences, Ulster University, Coleraine, UK

**Keywords:** multiparity, islet, islet morphology, beta-cell, cell lineage, transdifferentiation

## Abstract

**Background:**

Pregnancy induces a reversible expansion of pancreatic islet and beta-cell mass, but the impact of multiple pregnancies on these processes remains unclear.

**Methods:**

To further investigate this phenomenon, the current study employed transgenic models with beta- or alpha-cell lineage tracing capabilities, namely Ins1^*Cre/+*^ ;Rosa26-eYFP and Glu^*CreERT2*^;Rosa26-eYFP male mice, respectively. Using these models, we explored late-stage morphological islet adaptations and cellular plasticity in response to primi-, bi- and tri-parity.

**Results:**

All pregnant mice exhibited augmented islet and beta-cell areas, associated with decreased beta-cell apoptosis and increased proliferation. Notably, beta-cell proliferative capacity decreased as parity increased, but was still elevated in triparous mice when compared to null parous controls. Interestingly, alpha-cells also exhibited augmented growth and survival in all pregnant mice. In terms of cellular transdifferentiation, ductal to beta-cell conversion appeared greater in primiparous Ins1^*Cre/+*^;Rosa26-eYFP mice, but was much less obvious in bi- and tri-parous mice. Whilst quantification of beta- to alpha-cell transition events was more pronounced during pregnancy, it was less obvious in multiparity than primiparity. There were also notable reductions in beta-cell dedifferentiation, supporting positive effects of islet cell plasticity towards retention and expansion of beta-cell mass in multiparity. In harmony, alpha- to beta-cell transdifferentiation appeared markedly increased in multiparous Glu^*CreERT2*^ ;Rosa26-eYFP mice, coupled with augmented alpha-cell neogenesis and dedifferentiation, suggesting that these cells act as a principal source for beta-cell expansion.

**Conclusion:**

Together, these findings indicate that reduced beta-cell proliferation in multiparity is offset by enhanced islet cell plasticity, contributing to sustained islet adaptation across multiple gestations.

## Plain Language Summary

Effects of multiple pregnancies on pancreatic islets

Pregnancy is known to increase the size and function of insulin-secreting beta-cells of the pancreas. The mechanisms involved are not well known, and moreover, the impact of multiple pregnancies on this process is largely unexplored. We have used special mice with fluorescent labels within their beta-cells to investigate the impact of one, two and three pregnancies on islet and beta-cell adaptations in pregnancy. We have discovered that the ability of beta-cells to grow and proliferate is reduced as pregnancy number increases, but that islets and beta-cells are still enlarged despite this. Thus, our work has highlighted the ability of other cells within pancreatic islets to change their lineage, or type, and convert themselves into insulin producing beta-cells. We propose that this process, known as islet cell transdifferentiation, is critically important for maintaining islet size and function during second and third pregnancies. We also believe that problems with conversion of islet cells towards beta-cells, islet cell transdifferentiation, may lead to increased levels of diabetes that are noted in mothers who have given birth multiple times.

## Introduction

Pregnancy is accompanied by a well-documented expansion of pancreatic beta-cell mass, being apparent in both animals,^1^ and humans.^2^ This phenomenon is understood to be linked to increased insulin resistance during gestation,^3^ necessitating enhanced insulin secretion to maintain normoglycemia for both mother and fetus.^4^ Intriguingly, such adaptive changes in pancreatic islet morphology are transient, with beta-cell mass returning to pregestational levels following parturition.^5^ Thus, pregnancy exerts significant stress on beta-cells, both in terms of inducing rapid cellular expansion alongside a subsequent prompt regression of beta-cell mass. Moreover, there is suggestion that beta-cells become more glucose-responsive during pregnancy,^4^ placing further metabolic demand on these cells. When viewed in the context that beta-cells are one of the longest-lived cell types in the body,^6^ yet highly susceptible to oxidative stress,^7^ it is clear why pregnancy predisposes to gestational diabetes and that multiple pregnancies may increase the risk of diabetes.^8,9^

Whilst underlying mechanisms of pregnancy-induced changes in pancreatic beta-cell mass have been studied in some detail,^10^ there is still no conclusive appreciation of the cellular events involved. That said, we have recently demonstrated that beta-cell proliferation and survival, alongside increased transdifferentiation of islet endocrine and ductal cells into insulin-producing phenotypes, play integral roles in gestational beta-cell mass augmentation.^11^ These findings underscore the importance of cell plasticity towards the maintenance of normal pancreatic islet morphology, as well as in the pathogenesis of diseases associated with beta-cell decline, such as diabetes.^12^ Interestingly, the metabolic stress associated with multiparity, incorporating repeated enlargement and diminution of beta-cell mass, is believed to elevate the risk to postpartum diabetes,^13,14^ and has recently been suggested to be linked to impaired proliferative capacity of pancreatic beta-cells.^9^ However, there are no observations on the potential impact of multiparity on islet cell plasticity, which is noteworthy given the importance of this process on overall islet structure in health and disease.^15^

To address this gap, the current study has utilised lineage tracing technologies offered through fully characterised transgenic Ins1^*Cre/+*^;Rosa26-eYFP mice that permit for tracing of beta-cell identity over time,^12^ to investigate changes in beta-cell plasticity and overall pancreatic islet morphology following induction of multiple pregnancies. Thus, islets were studied from virgin female Ins1^*Cre/+*^;Rosa26-eYFP mice, as well as primiparous, biparous and triparous counterparts. Parallel observations pertaining to changes in alpha-cell lineage were also evaluated in related transgenic Glu^*CreERT2*^;Rosa26-eYFP mice.^16^ This is the first study of its kind to examine the impact of multiparity on islet cell transdifferentiation using such transgenic models, providing a comprehensive appreciation of overall islet cell plasticity events. Our work demonstrates a small reduction of beta-cell proliferative capacity in bi- and tri-parous mice when compared to primiparous mice, together with substantial and hitherto unsuspected changes in islet cell plasticity and neogenesis with pregnancy, that are dependent on parity.

## Materials and Methods

### Animals

Transgenic Ins1^*Cre/+*^;Rosa26-eYFP mice were bred and housed at Biomedical and Behavioural Research Unit at Ulster University, Coleraine, UK. The generation and characteristics of these mice have been described elsewhere.^17^ Additional observations, specifically on alpha-cell lineage, were made in age-matched female transgenic Glu^*CreERT2*^;Rosa26-eYFP mice.^18^ The transgene within pancreatic beta-cells of Ins1^*Cre/+*^;Rosa26-eYFP mice is constitutively active, whereas in the alpha-cells of Glu^*CreERT2*^;Rosa26-eYFP mice the transgene is tamoxifen-inducible, with these mice receiving 7 mg/mouse tamoxifen 7 days prior to culling to induce alpha-cell specific GFP expression.^19^ To provoke pregnancy, female mice were paired with males and closely monitored. All primiparous mice were 12 to 14 weeks of age, biparous mice 21 to 24 weeks old and triparous mice 32 to 38 weeks of age. All mice were housed in a temperature-controlled environment (22°C ± 2°C) on a 12-hour light/dark cycle. They were maintained on standard rodent maintenance chow (Trouw Nutrition, Norwich, UK) and normal drinking water *ad libitum*. All experiments were approved by Ulster University Animal Ethics Review Committee as well as under a UK Home Office Animal project license number PPL2902 (approved on April 26, 2021), conducted in accordance with the UK Animals (Scientific Procedures) Act 1986, and reported in line with the ARRIVE (Animal Research: Reporting of In Vivo Experiments) guidelines. Animals were age and weight matched for grouping, with no other inclusion/exclusion criteria applied.

### Immunohistochemistry

Mice were euthanised by lethal inhalation of CO_2_ followed by cervical dislocation within 4 weeks of giving birth. Immediately after excision, pancreatic tissue was fixed in 4% paraformaldehyde for 48 hours. Following fixation, tissue samples underwent dehydration and clearing before embedding in paraffin and sectioning (5 µm) for immunohistochemistry analysis, as described previously.^20^ Briefly, slides were immersed in xylene to remove wax, rehydrated in a series of ethanol washes of reducing concentration (100%-50%) followed by phosphate buffered saline (PBS). Antigen retrieval was achieved by immersion in heated citrate buffer (90°C, pH6) followed by blocking in 4% bovine serum albumin (BSA). Primary antibodies, including insulin, glucagon, GFP, Ki-67 and CK-19 were then added followed by appropriate secondary antibodies (Table 1), as described previously.^11^ Finally, slides were exposed to DAPI to identify nuclei before washing in PBS and mounting with glass coverslips. For assessing apoptosis, commercially available TUNEL staining (Roche Diagnostics, UK) was carried out following the manufacturer’s guidance. Stained slides were imaged on an Olympus BX-51 fluorescent microscope fitted with DAPI (350 nm), TRITC (594 nm) and FITC (488 nm) filters.

**Table 1.** Target, Species, Dilution and Source of Primary and Secondary Antibodies.

Target	Species	Dilution	Source
Primary antibodies			
Insulin	Mouse	1/400	Abcam; ab6995
Glucagon	Rabbit	1/100	Abcam; ab92517
Glucagon	Guinea pig	1/400	Raised in-house (PCA2/4)
Ki-67	Rabbit	1/500	Abcam; ab15580
GFP	Goat	1/500	Abcam; ab5450
CK-19	Rabbit	1/500	Abcam; ab76539
Secondary antibodies			
Mouse	Donkey	1/400	Invitrogen; A-21203
Rabbit	Donkey	1/400	Invitrogen; A-21206
Guineapig	Goat	1/400	Invitrogen; A-11073
Goat	Donkey	1/400	Invitrogen; A-11055

### Image Analysis

Cell^F^ imaging software (Olympus Soft Imaging Solutions) was employed to assess islet morphology as well as cellular proliferation, apoptosis, dedifferentiation, transdifferentiation and neogenesis, with blinded analysis of all images. Slides stained for insulin and glucagon were used to assess basic islet morphology and quantified on ImageJ software using a “closed polygon” tool, using the entire insulin positive islet area to quantify beta-cell area, glucagon positive islet area to measure alpha-cell area and combing these 2 values yielded total islet area in μm.^21^ Slides stained with TUNEL or Ki-67 were used to assess apoptosis and proliferation, respectively, in either insulin or glucagon positive cells, with apoptosis and proliferation rates quantified by calculating the number of alpha- or beta-cells expressing TUNEL or Ki-67, as appropriate, as a percentage of total alpha- or beta-cell numbers. Similarly, ductal cell transdifferentiation was quantified by assessing the percentage of CK-19 positively stained pancreatic ductal cells co-expressing insulin. For islet endocrine lineage analysis, in transgenic Ins1^*Cre/+*^;Rosa26-eYFP mice, GFP-positive cells are always of initial beta-cell lineage. Thus, islet cells expressing both insulin and GFP (insulin(+) GFP (+)) are considered original beta-cells, whereas cells positive for GFP but lacking insulin (insulin(−) GFP (+)) are dedifferentiated beta-cells, whilst islet cells expressing insulin without GFP (insulin(+) GFP (−)) are regarded as new beta-cells from a non-beta-cell source. Similarly, in Glu^*CreERT2*^;Rosa26-eYFP mice, GFP-positive cells are always of initial alpha-cell lineage. Therefore, for the current study islet cells positive for GFP and insulin (insulin(+) GFP (+)) are new beta-cells from an alpha-cell source, cells positive for GFP but lacking glucagon (glucagon(−) GFP (+)) are dedifferentiated alpha-cells and finally cells positive for glucagon but lacking GFP (glucagon(+) GFP (−)) are deemed as newly derived alpha-cells. For each parameter assessed, >60 islets were analysed per treatment group (n = 4-5).

### Statistical Analyses

Results were analysed using GraphPad PRISM (version 8), with data presented as mean ± SEM. Comparative analyses between the 2 groups of mice were carried out using a student’s t test. Results were deemed significant once *P* < .05. Data present biological replicates, which is a common practice for islet biology research since every islet possesses a significant inherent degree of heterogeneity. In terms of sample size calculations, at least 60 islets were examined for every parameter assessed, meaning the central limit theorem allows for the application of normal distribution-based statistical methods.

## Results

### Increased Parity Was Associated With Increased Islet and Beta-Cell Area

**Figure 1.**
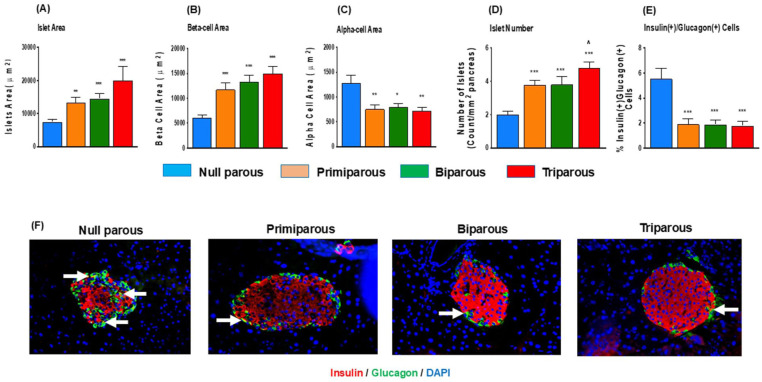
Impact of parity on pancreatic islet morphology in primiparous, biparous, tri parous female Ins1^*Cre/+*^;Rosa26-eYFP mice. Immunohistochemistry was conducted to assess (A) islet area, (B) beta-cell area, (C) alpha-cell area, (D) islet number as well as (E) double hormone positive islet cells. (F) Representative islet images showing insulin (red), glucagon (green) and DAPI (blue), with arrows depicting double hormone positive cells. Values are mean ± SEM (60 islets from n = 4-5 animals; at least 10 islets per mouse). Analysed using a one-way ANOVA with Bonferroni post hoc test with *P < .05, **P < .01 and ***P < .001 compared to null parous mice. ^^^*P* < .05 compared to primiparous mice.

Primiparity was associated with greater islet (*P* < .01) and beta-cell (*P* < .001) areas in transgenic Ins1^*Cre/+*^;Rosa26-eYFP mice (Figure 1A and B) with bi- and tri-parous mice exhibiting similar significant and numerically enhanced (*P* < .001) islet and beta-cell areas (Figure 1A and B). Interestingly, alpha-cell area was low (*P* < .05-.01) in all pregnant mice when compared to virgin controls (Figure 1C). In terms of islet number, increasing parity was associated with a substantially greater (*P* < .001) number of islets per mm^2^ of pancreas, with triparous mice exhibiting significantly more (*P* < .05) islets than primiparous mice (Figure 1D). Of note, all pregnant Ins1^*Cre/+*^;Rosa26-eYFP mice presented with lower (*P* < .001) numbers of islet cells co-expressing insulin and glucagon when compared to controls (Figure 1E). Figure 1F displays representative islet images stained for insulin and glucagon.

### Increased Parity Was Associated With Increased Alpha- and Beta-Cell Proliferation Whilst Apoptotic Rates of These Cells Are Suppressed

**Figure 2.**
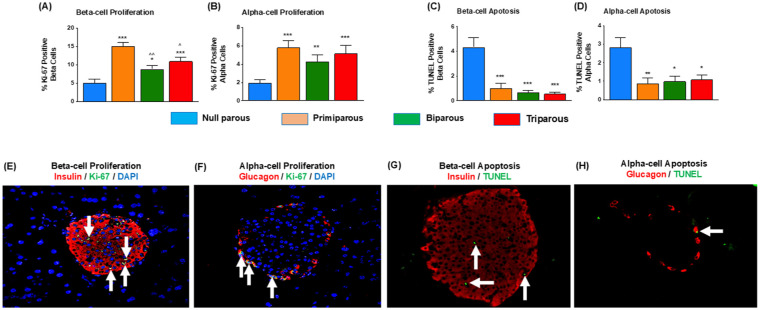
mpact of parity on islet alpha- and beta-cell proliferation and apoptosis in primiparous, biparous, tri parous female Ins1^*Cre/+*^;Rosa26-eYFP mice. Immunohistochemistry was conducted to assess (A) beta-cell proliferation, (B) alpha-cell proliferation, (C) beta-cell apoptosis and (D) alpha-cell apoptosis. (E-H) Representative images of beta-/alpha-cell proliferation and apoptosis, with arrows highlighting cells of interest. Values are mean ± SEM (60 islets from n = 4-5 animals; at least 10 islets per mouse). *P < .05, **P < .01 and ***P < .0001 compared to null parous mice. ^^^*P* < .05, ^^^^*P* < .01 compared to primiparous mice.

Primiparous Ins1^*Cre/+*^;Rosa26-eYFP mice exhibited greater (*P* < .001) alpha- and beta-cell proliferation rates when compared to control mice (Figure 2A and B). Alpha-cell proliferation was similarly enhanced (*P* < .01-.001) in biparous and triparous mice (Figure 2B). Whilst beta-cell proliferation was greater in (*P* < .05-.001) in biparous and triparous mice, it was still less (*P* < .05-.01) than in primiparous mice (Figure 2A). All pregnant mice had lower (*P* < .05-.001) rates of alpha- and beta-cell apoptosis in comparison to virgin controls (Figure 2C and D). Representative images displaying characteristic positive staining for insulin or glucagon in combination with either Ki-67 (Figure 2E and F) or TUNEL (Figure 2G and H) are also included.

### Increased Parity Was Associated With Reduced Beta-Cell Dedifferentiation, Augmented Beta- to Alpha-Cell Transdifferentiation but Has Differential Effects on Beta-Cell Neogenesis

**Figure 3.**
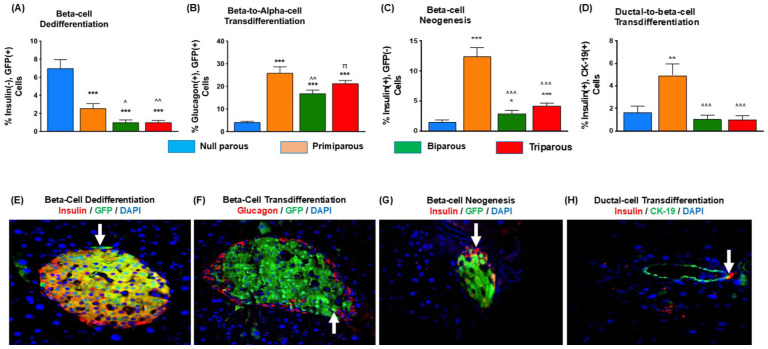
Impact of parity on beta-cell dedifferentiation, beta- to alpha-cell transdifferentiation, beta-cell neogenesis and ductal-to-beta-cell transdifferentiation in primiparous, biparous, tri parous female Ins1^*Cre/+*^;Rosa26-eYFP mice. Immunohistochemistry was conducted to assess (A) beta-cell dedifferentiation and (B) beta- to alpha-cell transdifferentiation, (C) beta-cell neogenesis and (D) ductal-to-beta-cell transdifferentiation. (E-H) Representative images showing (E,F,G) GFP and (H) CK-19 staining, highlighting positive co-staining, as appropriate. Values are mean ± SEM (60 islets from n = 4-5 animals; at least 10 islets per mouse). Analysed using a one-way ANOVA with Bonferroni post hoc test with *P < .05, **P < .01,***P < .001 compared to null parous mice, ^^^*P* < .05, ^^^^*P* < .01, ^^^^^*P* < .001 compared to primiparous mice, ^π^*P* < .05 compared to biparous mice.

Beta-cell dedifferentiation, characterised in Ins1^*Cre/+*^;Rosa26-eYFP by original GFP positive beta-cells no longer staining positive for insulin, was lower (*P* < .001) in primiparous mice when compared to null parous controls (Figure 3A). Bi- and tri-parous mice exhibited further lowering in beta-cell dedifferentiation, being significantly (*P* < .05-.01) less compared to primiparous mice (Figure 3A). However, beta- to alpha-cell transdifferentiation was greater (*P* < .001) in all pregnant mice (Figure 3B). Interestingly, beta- to alpha-cell transdifferentiation was lower (*P* < .01) in biparous mice when compared to primiparous mice, but elevated (*P* < .01) in triparous mice when compared to biparous mice (Figure 3B). The generation of new beta-cells from endocrine and non-endocrine sources (insulin(+) GFP(−) and insulin(+) CK19(+) stained cells; respectively) was substantially greater in primiparous mice when compared to controls (Figure 3C and D). Whilst bi- and tri-parous mice also had elevated (*P* < .05 and *P* < .001; respectively) beta-cell neogenesis from an endocrine origin, this was dramatically less (*P* < .001) in comparison to primiparous mice (Figure 3C), with multiparity also returning ductal to beta-cell transdifferentiation events towards control levels (Figure 3D). Representative images relating to typical islet cell transition events are depicted within Figure 3E-H.

### Glu^CreERT2^;Rosa26-eYFP Mice Reveal Greater Alpha-Cell Dedifferentiation, Alpha- to Beta-Cell Transdifferentiation as well as Alpha-Cell Neogenesis With Increasing Parity

**Figure 4.**
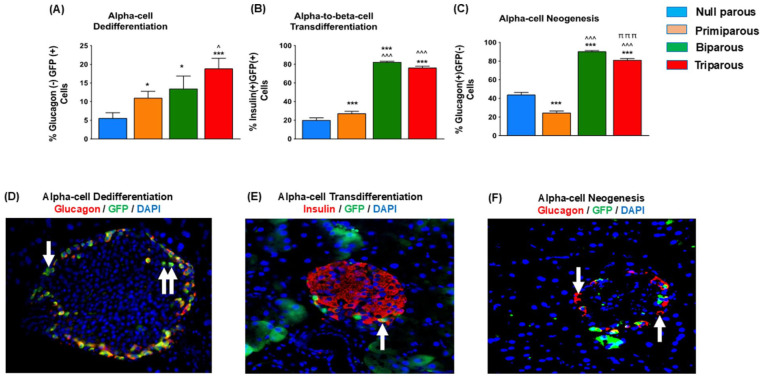
Impact of parity on alpha-cell dedifferentiation, alpha- to beta-cell transdifferentiation and alpha-cell neogenesis in primiparous, biparous, tri parous female Glu^*CreERT2*^;Rosa26-eYFP mice. Immunohistochemistry was conducted to assess (A) alpha-cell dedifferentiation (B) alpha- to beta-cell transdifferentiation and (C) alpha-cell neogenesis. (D-F) Representative images showing (D, F) glucagon (red), GFP (green) and DAPI (blue), as well as (E) insulin (red), GFP (green) and DAPI (blue) with arrows highlighting positive co-staining as appropriate. Values are mean ± SEM (60 islets from n = 4-5 animals; at least 10 islets per mouse). Analysed using a one-way ANOVA with Bonferroni post hoc test with *P < .05 and ***P < .001 compared to null parous mice, ^^^*P* < .05, ^^^^^*P* < .001 compared to primiparous mice, ^πππ^*P* < .001 compared to biparous mice.

Alpha-cell dedifferentiation, characterised by original GFP positive alpha-cells no longer staining positive for glucagon, was greater (*P* < .05-.001) as parity number increased (Figure 4A). This ultimately resulted in triparous mice presenting with more (*P* < .05) alpha-cell dedifferentiation events that primiparous mice (Figure 4A). In addition to this, primiparity was also associated with enhanced (*P* < .001) alpha- to beta-cell transdifferentiation but lower (*P* < .001) formation of new alpha-cells (Figure 4B and C). Bi- and tri-parity further augmented (*P* < .001) alpha- to beta-cell transdifferentiation (Figure 4B), and was also associated with elevated (*P* < .001) alpha-cell neogenesis (Figure 4C), with these effects being slightly less obvious in triparous Glu^*CreERT2*^;Rosa26-eYFP mice (Figure 4B and C). Representative images relating to typical islet cell transition events are depicted within Figure 4D-F.

## Discussion

Multiple pregnancies lead to recurring alterations of pancreatic islet beta-cell mass, to help overcome pregnancy related insulin resistance.^8^ Whilst it is believed that successive metabolic adaptations with multiparity lead to a deterioration of glucose tolerance and increase the risk of postpartum diabetes,^9^ there remain many unanswered questions about islet adaptations in pregnancy.^22^ Underlying cellular and molecular mechanisms are not well understood, although recent studies in rodents point towards a decreased beta-cell proliferative capacity following multiple pregnancies,^9^ with several hormonal drivers. This results in potential failure to compensate for insulin resistance with increasing risk of gestational diabetes and later development of overt type 2 diabetes.

The current study supports greater proliferation rates of beta-cells in primiparous mice when compared to virgin controls, and a subsequent diminution of this process in bi- and tri-parous female mice. However, beta-cell proliferation was still significantly greater with multiparity when compared to null parous mice, suggesting additional mechanisms contribute to pancreatic islet mass and adaptations associated with multiple pregnancies. It may also be worth noting that beta-cell proliferation rates appeared relatively heightened in virgin control mice, which may be related to the transgenic model employed.^23,24^ Beta-cell apoptosis was generally suppressed in all pregnant mice, but unlike primiparous mice,^11^ increased parity decreased utilisation of non-endocrine pancreatic ductal cells as a beta-cell source.^25,26^ This is consistent with previous studies indicating lesser importance of duct cells in the heightened beta-cell mass and function that are apparent during pregnancy.^27^

A possible central role of changes in cellular plasticity for regulating pancreatic islet morphology in pregnancy has not been examined until recently.^11^ In the present study, Ins1^*Cre/+*^;Rosa26-eYFP primiparous mice exhibited increased numbers of insulin-positive cells not expressing GFP, in agreement with our earlier observations.^11^ In addition, we observed that new beta-cells can originate from non-beta-cell endocrine sources in pregnancy.^15^ Interestingly, both these islet cell transitional effects appeared to wane with increased parity, similar to our findings with ductal to beta-cell transdifferentiation. In Ins1^*Cre/+*^;Rosa26-eYFP mice, pregnancy was also linked to an augmentation of the conversion of adult beta-cells to an alpha-cell phenotype. However, whilst beta- to alpha-cell transdifferentiation does occur in normal mice and may be heightened with pregnancy, such observations, which occur less frequently with multiparity, must be interpreted in light of the inherently beta-cell-rich architecture of murine islets.^28,29^ When viewed alongside a predisposition for retention of beta-cell identity, together with substantially decreased cellular apoptosis rates in multiparity, it does provide some explanation for increased islet number and beta-cell area in these mice. Of interest, higher parity has recently been associated with cellular ageing and increased risk of adverse cardiac remodelling,^30^ alluding to physiological disadvantages of multiparity.^31^ Whilst the current dataset provide evidence for enhanced beta-cell mass in multiparity that might initially appear advantageous, further studies are required to assess secretory function of these cells and the overall impact on metabolism. Thus, increasing beta-cell mass can be characteristic of unchecked insulin resistance.^32^ That said, current observations are made in mice during the early post-gestational stage, where changes in metabolic flux may be occurring,^30^ that would also need to be considered.

Related studies in Glu^*CreERT2*^;Rosa26-eYFP mice endorse islet alpha-cells as the likely chief origin of new beta-cells in biparous and triparous mice, although we are unable to totally exclude transdifferentiation of other endocrine cells such as delta- and PP-cells towards a beta-cell phenotype.^23,33^ Notwithstanding this, earlier work suggests that alpha- to beta-cell transdifferentiation exerts only a modest impact on beta-cell mass expansion in primiparous mice despite simultaneous observations of increased islet cell numbers in a transitional identity stage,^34^ potentially indicating that parity number or the timing of observations during the gestational period could be critical in terms of assessing islet cell plasticity during pregnancy. As such, our observations in primiparous Glu^*CreERT2*^;Rosa26-eYFP mice are somewhat similar to Szlapinski and co-workers,^27,34^ with less emphasis on changes in islet cell plasticity towards pregnancy-induced elevated beta-cell mass in first time mothers.

More notably however, islet cell transition events increased substantially with parity in Glu^*CreERT2*^;Rosa26-eYFP mice. In harmony, alpha-cell neogenesis and dedifferentiation were also elevated in bi- and tri-parous mice, alongside a consistent increase in alpha-cell proliferation and decrease of related apoptosis in pregnant Ins1^*Cre/+*^;Rosa26-eYFP, independent of parity number. Thus, there is a growing awareness that alpha-cells can act as a progenitor pool for beta-cells through the process of islet endocrine cell transdifferentiation,^15^ which seems to be of relevance in the islet adaptations that accompany multiparity and in keeping with no associated increase in alpha-cell area in these mice. In brief, at first glance, changes in islet cell turnover rates in all pregnant mice, that included elevated proliferation and cell survival rates, would instinctively point towards augmentation of both alpha- and beta-cell areas, but this was not the case for the alpha-cell population. Thus, increased rates of islet cell transition events, seemingly alpha- to beta-cell transdifferentiation, and especially with multiparity, led to prominently elevated islet and beta-cell areas, with subsequent reductions of alpha-cell area. Although outside the scope of the current study, an understanding of related islet cell transition events during subsequent regression of beta-cell mass in consecutive pregnancies would also be of interest,^35^ and may help further uncover the role of islet cell plasticity in islet adaptations linked to multiple pregnancies. In addition, it would also be informative to uncover whether transitioning islet cells pass through an embryonic phase in Ins1^*Cre/+*^;Rosa26-eYFP and Glu^*CreERT2*^;Rosa26-eYFP mice,^15^ and how this then might be affected by multiple pregnancies. Furthermore, translation of our findings to the human setting, where the molecular mechanisms that drive pregnancy-induced islet adaptations may differ from those in mice,^36^ requires consideration.

## Conclusion

Multiparous pregnancy in Ins1^*Cre/+*^;Rosa26-eYFP transgenic mice is associated with lower beta-cell proliferative capacity, but importantly only when compared to primiparous mice. Furthermore, using 2 separate transgenic mouse models with islet cell tracing capabilities, we have shown that alterations of islet cell plasticity are integral for adaptions of pancreatic islet morphology in pregnancy, and particularly with multiparity. Additional studies to determine the functionality of beta-cells that have transitioned from other islet cell types may help uncover the overall impact of islet cell plasticity on metabolic control during pregnancy. However, the current study clearly points towards a previously unappreciated role of changes in islet cell lineage supporting adaptations of pancreatic islet morphology in multiparity.
